# Global disparities in scientific publications: A 5-year analysis of 10 critical care journals

**DOI:** 10.1016/j.ccrj.2025.100137

**Published:** 2025-10-16

**Authors:** R. Daltro-Oliveira, A. Quintairos, L.I.O. Santos, F. Amado, J.I.F. Salluh, A.P. Nassar

**Affiliations:** a Fundação Antonio Prudente- A C Camargo Cancer Center, São Paulo, SP, Brazil; b Adult ICU, Grupo Santa Joana, São Paulo, SP, Brazil; c General ICU, Hospital Vila Nova Star – Rede D’Or, São Paulo, SP, Brazil; d Department of Critical Care and Postgraduate Program in Translational Medicine, D'Or Institute for Research and Education, Rio de Janeiro, RJ, Brazil; e Department of Critical Care Medicine, Mater Dei Porto Dias Hospital, Belém, Pará, Brazil

**Keywords:** Critical care, Scientific publishing, Global health disparities, Low- and middle-income countries

## Abstract

**Objective:**

To evaluate the global distribution of original research articles in intensive care journals, analysing differences by country income level and assessing study characteristics, including type, funding, and accessibility.

**Design:**

A retrospective bibliometric analysis of original research articles published between 2018 and 2022.

**Setting:**

The 10 intensive care journals with the highest 2022 Impact Factors, as identified by Clarivate Analytics.

**Participants:**

Original research articles (observational studies and randomised controlled trials) in adult intensive care, excluding paediatric and preclinical studies.

**Main outcome measures:**

Country of affiliation of the corresponding author (classified by World Bank income level), study type, funding source, number of participating centres, open-access status, and thematic category.

**Results:**

Among 12,441 manuscripts reviewed, we included 4982 original research articles. Of these, 4479 (89.9 %) were from high-income countries (HICs), 446 (9.0 %) from upper-middle-income countries (UMICs), 53 (1.1 %) from lower-middle-income countries (LMICs), and 4 (0.1 %) from low-income countries (LICs). Overall, 434 (8.7 %) were randomised controlled trials, with higher proportions in UMICs (13.2 %) and LMIC (20.8 %) studies compared with HICs (8.1 %). Multicenter design was reported in 44.6 % of all studies, but less frequently in UMICs (29.1 %) and LMICs (30.2 %). The median sample size was 228 patients (interquartile range, 76-1052), ranging from 231 in HICs to 211 in UMICs and 100 in LMICs. Funding was reported in 56.9 % of studies, most often from public sources. Public funding supported 28.0 % of UMICs and 9.4 % of LMICs studies, compared with 14.3 % of HIC studies. Open-access articles represented 44.8 % overall and were more common among funded studies.

**Conclusions:**

Publications from UMICs, LMICs, and LICs remain underrepresented in high-impact intensive care journals. Structural barriers, limited funding, and potential publication bias contribute to this imbalance. Addressing these gaps requires greater funding opportunities, equitable collaborations, and stronger editorial commitment to inclusivity.

## 1 Introduction

Over 1.1 million scientific papers are published annually, with approximately 12,000 focusing on intensive care.^1^ However, most biomedical scientific production is concentrated in high-income countries (HICs). Evaluating the five highest-impact journals in general medicine, Woods et al. showed in 2004 and again in 2023 that HICs are responsible for almost 90 % of publications in these journals.^2^ More recently, Charpignon et al. revealed that among authors publishing in high-impact general medical journals, 87.5 % were affiliated with HIC institutions, while only 10.4 % of papers included at least one author affiliated with low- and middle-income countries.^3^ By cross-referencing data from SciMago and the World Bank’s geo-economic classifications, our group demonstrated that between 2018 and 2022, 77.9 % of papers published in intensive care originated from HICs, even though only a quarter of the world’s population resides in these countries.^4^

Such disparity in scientific output has significant implications for healthcare. Even more than 20 years after its publication, the 10/90 gap—which states that “only about 10 percent of the global biomedical research budget is allocated to diseases accounting for roughly 90 percent of the world's health problems”—remains relevant.^5^ Evidence suggests that research funding is disproportionately directed toward conditions more prevalent in high-income countries, often neglecting conditions that pose a greater global burden.^6^

Despite these concerns, few studies have explored how critical care publications are distributed across income groups. The present study aims to address this gap by analysing original research publications in critical care journals between 2018 and 2022. Specifically, we aim to describe how scientific publications vary by country income level and to examine the aspects of these studies, including study type and funding sources.

## 2 Methods

We evaluated all citable documents published between 2018 and 2022 in the top 10 journals with the highest impact factors for 2022 as reported by Clarivate Analytics:^7^ Intensive Care Medicine, Critical Care, Critical Care Medicine, Annals of Intensive Care, Journal of Intensive Care, Journal of Critical Care, Neurocritical Care, Critical Care and Resuscitation, Journal of Intensive Care Medicine, and Australian Critical Care. We included only journals exclusively dedicated to intensive care, excluding those that did not publish original research (e.g. review-only journals) or covered intensive care alongside other medical specialities.

We reviewed each issue of these journals to identify original research studies, i.e. observational (cohort, case–control, and transversal) and randomised controlled trials (RCTs). Thus, we excluded letters, comments, images, systematic reviews, narrative reviews, scoping reviews, and other non-original publications. Additionally, we excluded studies focused on paediatric intensive care and preclinical research with a laboratory focus from the pool of original research articles.

We categorised all manuscripts by the income level of the country where the corresponding author was affiliated. We used the World Bank classification to define the countries as high-income countries (HICs), upper-middle-income countries (UMICs), lower-middle-income countries (LMICs), and low-income countries (LICs).^8^ In this framework, the term middle-income countries (MICs) encompasses both UMICs and LMICs.

We evaluated the following criteria: publication year, study type (observational vs. RCT), country of affiliation of the corresponding author, income level of the country (as classified by the World Bank), study theme (categorised in sepsis/infection, hemodynamics/cardiovascular, mechanical ventilation/respiratory, renal/dialysis, neurocritical care, sedation/delirium, COVID-19, nutrition, and quality/organisation), number of participating centres, and open-access status. For studies where the corresponding author had multiple affiliations in different countries, we determined the primary affiliation based on the listed correspondence address or the email domain associated with a specific institution. Only articles explicitly classified as open access by the publisher were considered, regardless of institutional access options.

To assess the diversity of each journal, we quantified the number of different countries that published articles during the study period. We further examined publication concentration by calculating the proportion of total publications contributed by the top 3 and top 10 countries.

We recorded data in Microsoft Excel (Microsoft Corporation, Redmond, WA, USA), processed and analysed it in R Studio (RStudio, PBC, Boston, MA, USA). We visualised it using Tableau Public (Tableau Software, Seattle, WA, USA). We present all categorical variables as absolute numbers and proportions and all numeric variables as medians and interquartile ranges (IQRs).

## 3 Results

**Fig. 1:**
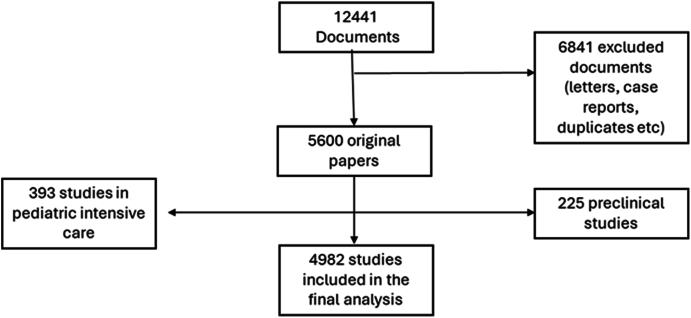
Flowchart of article selection.

We evaluated a total of 12,441 manuscripts. Of these, 5600 were original research articles, including 393 studies in paediatric intensive care and 225 preclinical studies. The final analysis included 4982 original research articles (Fig. 1).

**Fig. 2:**
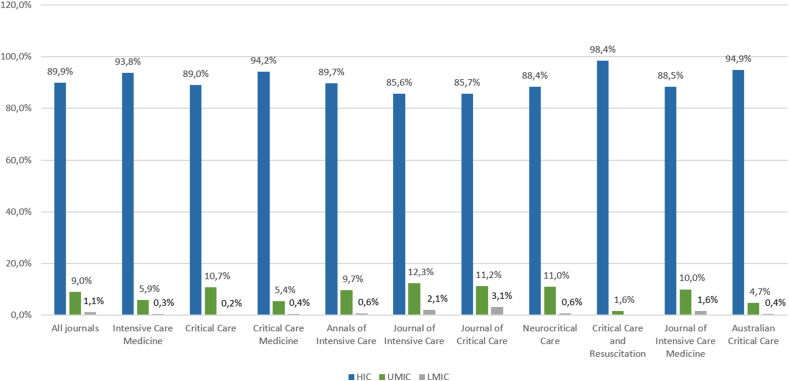
Proportion of original research articles from high-income countries (HICs), upper-middle-income countries (UMICs), and lower-middle-income (LMICs) across the 10 selected intensive care journals (2018-2022).

Among the 4982 selected studies, 4479 (89.9 %) were from HICs, 446 (9.0 %) from UMICs, 53 (1.1 %) from LMICs, and 4 (0.1 %) from LICs. This distribution was consistent across all journals (Fig. 2).

Overall, only 8.7 % (n = 434) were RCTs; however, this proportion was higher in UMIC studies (59 [13.2 %]) and LMIC studies (11 [20.8 %]) compared with HICs (364 [8.1 %]). After considering all the studies, 2224 (44.6 %) were multicenter, with lower proportions in UMIC (153 [29.1 %]) and LMIC (16 [30.2 %]) studies than in HICs (2055 [45.9 %]). The median sample size across all studies was 228 patients (interquartile range [IQR], 76-1052). When stratified by income level, the median sample size was 231 (IQR, 75-1071) for HICs, 211 (IQR, 87-802) for UMICs, and 100 (IQR, 65-249) for LMICs (Table 1). A more detailed description of the evaluated articles, stratified by journal and by the income level of the corresponding author's country, is available in Supplementary Table 1.

**Table 1:** Study characteristics of original research articles published in the 10 highest-impact intensive care journals (2018-2022), stratified by income level.

Features	All N = 4,978^a^	HIC N = 4,479^a^	UMIC N = 446^a^	LMIC N = 53^a^	LIC N = 4^a^
Study type					
Observational	4544 (91.3 %)	4115 (91.9 %)	387 (86.8 %)	42 (79.2 %)	4 (100 %)
RCT	434 (8.7 %)	364 (8.1 %)	59 (13.2 %)	11 (20.8 %)	0
Multicenter	2224 (44.6 %)	2055 (45.9 %)	153 (29.1 %)	16 (30.2 %)	3 (75 %)
Open-acess	2232 (44.8 %)	2014 (45.0 %)	202 (45.3 %)	16 (30.2 %)	0
Sample size	228 (76, 1052)	231 (75, 1071)	211 (87, 802)	100 (65, 249)	122.5 (13.5, 294.5)
Funding					
Public	1501 (30.2 %)	1245 (27.8 %)	245 (54.9 %)	11 (20.7 %)	1 (25 %)
Private	274 (5.5 %)	268 (6.0 %)	6 (1.4 %)	0	1 (25 %)
Non-profit	244 (4.9 %)	229 (5.1 %)	12 (2.7 %)	3 (5.7 %)	0
Mixed	817 (16.4 %)	767 (17.1 %)	47 (10.5 %)	3 (5.7 %)	1 (25 %)
No funding	1579 (31.7 %)	1436 (32.1 %)	113 (25.3 %)	30 (56.6 %)	0
No data	563 (11.3 %)	534 (11.9 %)	23 (5.2 %)	6 (11.3 %)	1 (25 %)

HICs, High-Income Level Countries; UMICss, Upper-Middle-Income Level Countries; LMICs, Lower-Middle-Income Level Countries; LICs, Low-Income Level Countries; RCT, Randomised Clinical Trial.

a n (%); Median (Q1, Q3).

Regarding funding, 2836 (56.9 %) studies reported financial support, with public funding being the most frequent source. Public funding accounted for 125 (28.0 %) of all studies from UMICs and 5 (9.4 %) from LMICs, compared with 642 (14.3 %) of those from HICs. The open-access status accounted for 2232 manuscripts (44.8 %) and was comparable across income levels (Table 1). When stratified by funding type, we observed a lower prevalence of open-access manuscripts among those that either did not receive funding or did not report funding information. This trend was more pronounced for HIC studies compared with UMIC and LMIC studies (Table 2).

**Table 2:** Proportion of open-access publications according to funding source and income level of the corresponding author’s country.

Funding	Income level		Open-access
Mixed	HIC	N = 767	360 (46.9 %)^a^
	UMIC	N = 47	20 (42.6 %)^a^
	LMIC	N = 3	2 (66.7 %)^a^
	LIC	N = 1	0
No funding	HIC	N = 1436	628 (43.7 %)^a^
	UMIC	N = 113	44 (38.9 %)^a^
	LMIC	N = 30	8 (26.7 %)^a^
	LIC	N = 0	0
No data	HIC	N = 534	130 (24.3 %)^a^
	UMIC	N = 23	4 (17.4 %)^a^
	LMIC	N = 6	0
	LIC	N = 1	0
Non-profit	HIC	N = 229	105 (45.9 %)^a^
	UMIC	N = 12	7 (58.3 %)^a^
	LMIC	N = 3	1 (33.3 %)^a^
	LIC	N = 0	0
Private	HIC	N = 268	149 (55.6 %)^a^
	UMIC	N = 6	2 (33.3 %)^a^
	LMIC	N = 1	0
	LIC	N = 1	0
Public	HIC	N = 1245	642 (51.6 %)^a^
	UMIC	N = 245	125 (51.0 %)^a^
	LMIC	N = 11	5 (45.5 %)^a^
	LIC	N = 1	0

HICs, High-Income Level Countries; UMICs, Upper-Middle-Income Level Countries; LMICs, Lower-Middle-Income Level Countries; LICs, Low Income Level Countries.

a n (%).

**Fig. 3:**
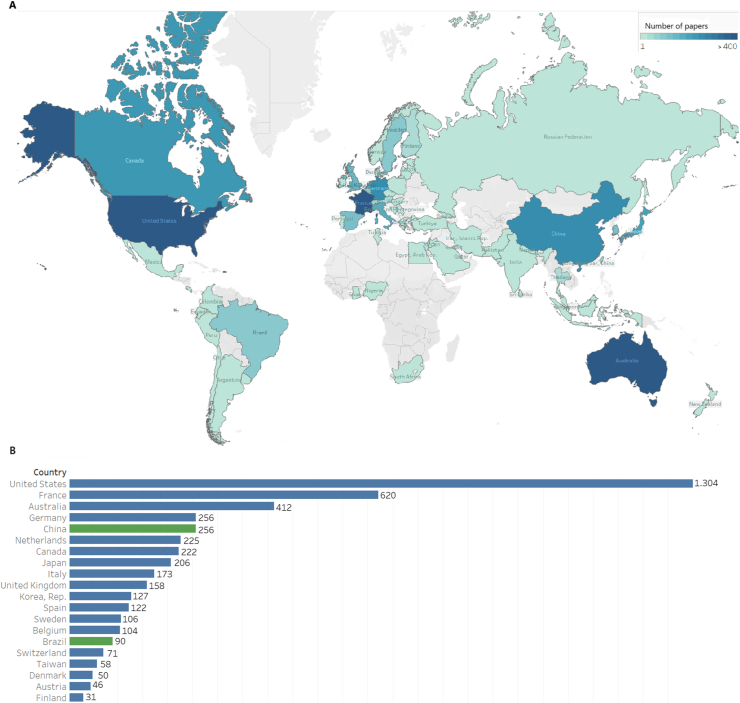
Geographic distribution of critical care publications (2018-2022). A, A world map showing the number of publications by country. B, Bar chart of the top 20 publishing countries.

The United States was the leading publisher of studies during the period, with nearly twice as many publications as that of France. Among the 20 largest publishing countries, only two were MICs (China and Brazil) (Fig. 3A). Fig. 3B presents a heat map illustrating the geographic distribution of intensive care publications.

**Fig. 4:**
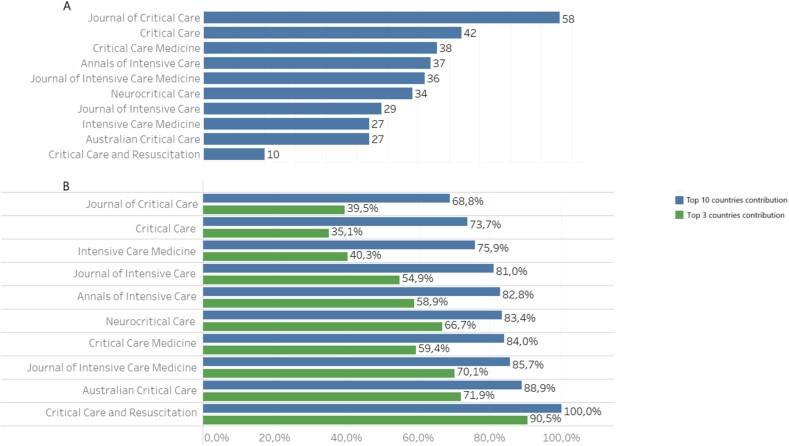
A, A number of different countries that published original research articles in each journal between 2018 and 2022. B, The proportion of articles published by the top 3 and top 10 most publishing countries in each journal.

The number of countries that published in each journal ranged from 10 to 58 (Fig. 4A). The top 3 countries accounted for 35.1 %-90.5 % of all publications in each journal, while the top 10 countries contributed between 68.1 % and 100 % (Fig. 4B).

Among the topics studied, sepsis/infection, hemodynamics/cardiovascular, and neurocritical care were the most frequently addressed (Table 3).

**Table 3:** Distribution of study topics in original research articles from high- and middle-income countries.

Characteristic	HIC N = 4,479^a^	UMIC N = 446^a^	LMIC N = 53^a^	LIC N = 4^a^
Topics				
COVID-19	300 (6.7 %)	44 (9.9 %)	0	0
Hemodynamics/cardiovascular	543 (12.1 %)	36 (8.0 %)	1 (1.9 %)	0
Mechanical ventilation/respiratory	375 (8.4 %)	50 (11.2 %)	8 (15.1 %)	0
Neurocritical care	718 (16.0 %)	69 (15.5 %)	4 (7.5 %)	2 (50.0 %)
Nutrition	84 (1.9 %)	11 (2.5 %)	3 (5.6 %)	0
Other	1064 (23.8 %)	62 (13.9 %)	12 (22.6 %)	0
Quality/organization	309 (6.9 %)	23 (5.2 %)	8 (15.1 %)	2 (50.0 %)
Renal/dialysis	224 (5.0 %)	36 (8.1 %)	2 (3.8 %)	0
Sedation/delirium	148 (3.3 %)	11 (2.5 %)	3 (5.6 %)	0
Sepsis/Infection	714 (15.9 %)	104 (23.3 %)	12 (22.6 %)	0

HICs, High-Income Level Countries; UMICs, Upper-Middle-Income Level Countries; LMICs, Lower-Middle-Income Level Countries; LICs, Low-Income Level Countries.

a n (%).

## 4 Discussion

Our study suggests that, as previously demonstrated in some medical specialities, original research articles from MICs and LICs are underrepresented in intensive care. This behaviour is more pronounced in journals of intensive care societies from HICs (intensive care medicine, critical care medicine, and critical care resuscitation), where the percentage of original publications from MICs and LICs can be as low as 1.6 %. Although no previous studies have shown this in intensive care, prior research has already highlighted the gap in scientific output between HICs and MICs, as well as the focus on diseases that more severely impact HICs.^6^ Moreover, evidence suggests this research imbalance has not improved substantially in recent years.^9^

The persistent inequality in scientific production poses a significant global health challenge in several ways.^4^^,^[10], [11], [12], [13] First, the lack of local and global research and monitoring of diseases affecting non-high-income countries weakens the world’s ability to detect, track, and respond effectively to increasingly frequent international health emergencies, as demonstrated with monkeypox, COVID-19, and Ebola. Without adequate surveillance and research in these regions, emerging health threats can go unnoticed until they escalate into global crises.^9^^,^^14^ Second, countries with different income levels face distinct health challenges and disease patterns. When research is concentrated mainly in HICs, it overlooks the unique health needs of other countries, leading to significant gaps in medical knowledge and hindering the development of relevant healthcare solutions. Third, the structural disparities among countries, particularly in intensive care—which demands highly specialised human and technological resources—make it challenging to apply knowledge generated in HICs to MICs or LICs, given the starkly different healthcare realities. Overall, this imbalance in scientific production plays a crucial role in perpetuating global health inequities; additionally, being involved in research may also contribute to improving ICU organization and, consequently, patient outcomes.^11^^,^^15^

Notably, the proportion of RCTs among MIC articles is higher than that of HIC articles. One possible explanation is that the higher proportion of RCTs among MIC publications may reflect a perception among researchers that stronger methodological designs are required to improve the likelihood of acceptance in high-impact intensive care journals which may in turn discourage MIC investigators from submitting their work to these journals. Another reason could be that RCTs from HICs are more often published in high-impact general medicine journals, which proportionally reduces their presence in specialised intensive care journals.

Moreover, we also found significant differences between HICs and MICs studies regarding funding. Studies from MICs rely much more heavily on public funding and are rarely supported by the private sector. This contrast becomes even more pronounced when we consider that HICs invest a larger share of their gross domestic product in research and development than MICs. In 2021, the disparity in research and development (R&D) investment between HICs and MICs was substantial, with HICs investing approximately 1.12 trillion USD more than MICs, according to World Bank data.^8^ The Global Observatory on Health Research and Development reported that, in recent years, only about 1 % of funding from the world’s 10 largest international health research funders has been allocated to research and development MICs. This also includes investments in priority areas identified by the World Health Organization, such as infectious diseases that disproportionately affect these countries, including malaria, dengue, Chagas disease, and leishmaniasis.^9^^,^^16^

This complex landscape of inequality extends beyond research development and funding—it also manifests in challenges related to publishing, especially within the open-access framework. The open-access model can make scientific content more accessible, particularly for researchers and institutions in MICs and LICs. However, in our study, more open-access publications from HICs report no funding compared to those from MICs. One possible explanation for this is the economic impact that Article Processing Charges (APCs) may have on authors. When adjusting for purchasing power parity, the burden of these fees is much greater for MIC authors.^13^ Additionally, the grants secured by publishers are more often directed towards LIC countries, which, as this study shows, contribute only a small number of these publications, making this funding benefit effectively negligible in practice.

Several other factors contribute to this imbalance in scientific production. HICs have better research infrastructure, with roughly twice as many health researchers (in full-time equivalent) per million inhabitants compared to MICs.^17^ Language is another barrier as nearly all high-impact journals in intensive care publish exclusively in English, despite the fact that only about 20 % of MICs have English as an official language.^12^

This study has several limitations. First, by focusing exclusively on original research articles and excluding systematic reviews or meta-analyses—which often require fewer resources—we may have underrepresented contributions from MICs and LICs. Systematic reviews and meta-analyses may, in fact, represent an attractive option for researchers from MICs and LICs to publish in high-impact journals, serving both as an academic strategy and as a potential means of career advancement. However, we decided to exclude this type of study to focus on original studies, whose workload tends to be greater, and which need to involve larger research groups and some degree of funding or structure, even if it comes from the researchers themselves or the institutions they are affiliated with. Thus, our approach underscores how economic disparities can shape the entire scientific production process of the original studies. Second, although LIC data were included in our analysis, their very limited representation remains a constraint and should be acknowledged as a limitation when interpreting our findings. Third, restricting the analysis to 10 intensive care journals improved feasibility but may have excluded a substantial proportion of relevant scientific content from regional or lower-impact journals and those addressing medical topics other than intensive care. However, we chose to select only the top 10 journals in the field to address the studies that the scientific community considered most impactful during that period. Finally, the 5-year timeframe limits our ability to assess longitudinal trends and increases susceptibility to external influences, such as the COVID-19 pandemic.

Addressing these disparities requires coordinated action from funding agencies, research institutions, and scientific journals. International consortia could prioritise programs that support investigators from non-HICs, while sustained collaborations with HIC partners may strengthen local research capacity and expand opportunities for multicenter studies. Importantly, journals also play a decisive role. Editorial boards should be sensitised to the importance of representing high-quality research from MICs and LICs, and wider adoption of mechanisms such as double-blind peer review could help mitigate implicit biases in the publication process. Beyond considerations of global health security, high-quality research from non-HICs deserves publication in its own right—because good science, regardless of origin, contributes to the collective advancement of knowledge. High-impact journals are not only the voices of their societies or regions but serve as platforms for humanity as a whole and ensuring that strong work from MICs and LICs is visible is essential to that mission.

## 5 Conclusion

In conclusion, our study shows that publications from MICs and LICs remain underrepresented in high-impact intensive care journals. Several factors may contribute to this inequality, including structural barriers, funding limitations, and potential publication bias. This imbalance reinforces global health disparities and limits the diversity of perspectives represented in scientific literature. Addressing these challenges will require not only increasing public and international funding for research from non-HICs and fostering equitable international collaborations but also greater commitment from journals. Editorial boards must recognise the value of high-quality science from all regions and adopt practices—such as broader representation and double-blind peer review—that promote fairness and inclusivity. Ensuring that strong research from MICs and LICs is visible is not only a matter of global health security but also a responsibility of the scientific community to uphold equity and advance knowledge for the benefit of humanity.

## CRediT authorship contribution statement

**Daltro-Oliveira R:** Investigation, Data Curation, Writing - Original Draft, Project administration, Formal analysis; **Quintairos A:** Investigation; **Santos LIO:** Investigation; **Amado F:** Investigation; **Salluh JIF:** Conceptualization, Writing - Review & Editing; **Nassar AP Jr:** Conceptualization, Writing - Review & Editing, Supervision.

## Funding

This research did not receive any specific grant from funding agencies in the public, commercial, or not-for-profit sectors.

## Declaration of Generative AI and AI-assisted technologies in the writing process

Statement: During the preparation of this work, the author(s) used ChatGPT to improve the readability and language of the manuscript. After using this tool/service, the author(s) reviewed and edited the content as needed and take(s) full responsibility for the content of the published article.

## Conflicts of interest

The authors declare the following financial interests/personal relationships which may be considered as potential competing interests: Jorge Ibrain Figueira Salluh reports a relationship with Conselho Nacional de Desenvolvimento Científico e Tecnológico that includes funding grants. Antonio Paulo Nassar Junior reports a relationship with Conselho Nacional de Desenvolvimento Científico e Tecnológico that includes funding grants. Jorge Ibrain Figueira Salluh reports a relationship with Fundação Carlos Chagas Filho de Amparo à Pesquisa do Rio de Janeiro that includes funding grants. If there are other authors, they declare that they have no known competing financial interests or personal relationships that could have appeared to influence the work reported in this paper.
