# Supplementary material for: Global disparities in scientific publications: A 5-year analysis of 10 critical care journals
Source: Crit Care Resusc. 2025 Oct 16;27(4):100137. doi: 10.1016/j.ccrj.2025.100137 (PMC12554101; doi:10.1016/j.ccrj.2025.100137)
Supplement: Multimedia component 1 [file mmc1.docx]

|  | All journals | | | INTENS CARE MED | | CRIT CARE | | | CRIT CARE MED | | | | | ANN INTENSIVE CARE | | | | | J INTENSIVE CARE | | | | | J CRIT CARE | | | | NEUROCRIT CARE | | | CRIT CARE RESUSC | | | | | | | J INTENSIVE CARE MED | | | | | AUST CRIT CARE | | |
| --- | --- | --- | --- | --- | --- | --- | --- | --- | --- | --- | --- | --- | --- | --- | --- | --- | --- | --- | --- | --- | --- | --- | --- | --- | --- | --- | --- | --- | --- | --- | --- | --- | --- | --- | --- | --- | --- | --- | --- | --- | --- | --- | --- | --- | --- |
| **Features** | **All** N = 4,978^1^ | **HIC**  N = 4,479^1^ | **MIC**  N = 499^1^ | **HIC**  N = 272^1^ | **MIC**  N = 18^1^ | **HIC**  N = 788^1^ | **MIC**  N = 97^1^ | | **HIC**  N = 837^1^ | | **MIC**  N = 52^1^ | | **HIC**  N = 437^1^ | | | **MIC**  N = 50^1^ | | **HIC**  N = 167^1^ | | | **MIC**  N = 28^1^ | | **HIC**  N = 742^1^ | | | **MIC**  N = 124^1^ | | **HIC**  N = 436^1^ | | **MIC**  N = 57^1^ | | | **HIC**  N = 124^1^ | | **MIC**  N = 2^1^ | | **HIC**  N = 453^1^ | | | **MIC**  N = 59^1^ | | **HIC**  N = 223^1^ | | | **MIC**  N = 12^1^ |
| Study type |  |  |  |  |  |  |  |  | |  | |  | | |  | |  | | |  | |  | | |  | |  | |  | | |  | |  | |  | | |  | |  | | |  | |
| Observational | 4,544 (91%) | 4,115 (92%) | 429 (86%) | 199 (73%) | 15 (83%) | 698 (89%) | 77 (79%) | 774 (92%) | | 41 (79%) | | 403 (92%) | | | 40 (80%) | | 158 (95%) | | | 25 (89%) | | 695 (94%) | | | 111 (90%) | | 422 (97%) | | 56 (98%) | | | 110 (89%) | | 2 (100%) | | 445 (98%) | | | 52 (88%) | | 211 (95%) | | | 10 (83%) | |
| RCT | 434 (8.7%) | 364 (8.1%) | 70 (14%) | 73 (27%) | 3 (17%) | 90 (11%) | 20 (21%) | 63 (7.5%) | | 11 (21%) | | 34 (7.8%) | | | 10 (20%) | | 9 (5.4%) | | | 3 (11%) | | 47 (6.3%) | | | 13 (10%) | | 14 (3.2%) | | 1 (1.8%) | | | 14 (11%) | | 0 (0%) | | 8 (1.8%) | | | 7 (12%) | | 12 (5.4%) | | | 2 (17%) | |
| Multicenter | 2,224 (45%) | 2,055 (46%) | 169 (34%) | 227 (83%) | 14 (78%) | 422 (54%) | 45 (46%) | 466 (56%) | | 25 (48%) | | 193 (44%) | | | 17 (34%) | | 77 (46%) | | | 5 (18%) | | 270 (36%) | | | 37 (30%) | | 145 (33%) | | 12 (21%) | | | 62 (50%) | | 2 (100%) | | 126 (28%) | | | 11 (19%) | | 67 (30%) | | | 1 (8.3%) | |
| Sample size | 228 (76, 1,052) | 231 (75, 1,071) | 211 (87, 802) | 710 (250, 2,466) | 3,439 (332, 129,680) | 241 (71, 1,167) | 252 (107, 1,813) | 393 (102, 3,603) | | 298 (111, 1,341) | | 159 (61, 582) | | | 208 (100, 500) | | 235 (85, 2,062) | | | 94 (60, 300) | | 248 (79, 1,094) | | | 223 (81, 1,190) | | 120 (48, 330) | | 186 (88, 317) | | | 187 (72, 606) | | 629,814 (49, 1,259,578) | | 207 (84, 783) | | | 126 (72, 348) | | 100 (40, 280) | | | 155 (101, 604) | |
| Open-acess | 2,232 (45%) | 2,014 (45%) | 218 (44%) | 75 (28%) | 4 (22%) | 788 (100%) | 97 (100%) | 133 (16%) | | 8 (15%) | | 437 (100%) | | | 50 (100%) | | 167 (100%) | | | 28 (100%) | | 80 (11%) | | | 11 (8.9%) | | 126 (29%) | | 9 (16%) | | | 124 (100%) | | 2 (100%) | | 60 (13%) | | | 8 (14%) | | 24 (11%) | | | 1 (8.3%) | |
| Funding |  |  |  |  |  |  |  |  | |  | |  | | |  | |  | | |  | |  | | |  | |  | |  | | |  | |  | |  | | |  | |  | | |  | |
| Public | 1,501 (30%) | 1,245 (28%) | 256 (51%) | 88 (32%) | 10 (56%) | 292 (37%) | 63 (65%) | 185 (22%) | | 22 (42%) | | 132 (30%) | | | 33 (66%) | | 45 (27%) | | | 18 (64%) | | 181 (24%) | | | 45 (36%) | | 134 (31%) | | 38 (67%) | | | 23 (19%) | | 0 (0%) | | 101 (22%) | | | 24 (41%) | | 64 (29%) | | | 3 (25%) | |
| Private | 274 (5.5%) | 268 (6.0%) | 6 (1.2%) | 22 (8.1%) | 0 (0%) | 56 (7.1%) | 0 (0%) | 65 (7.8%) | | 1 (1.9%) | | 28 (6.4%) | | | 0 (0%) | | 10 (6.0%) | | | 1 (3.6%) | | 36 (4.9%) | | | 2 (1.6%) | | 21 (4.8%) | | 2 (3.5%) | | | 3 (2.4%) | | 0 (0%) | | 24 (5.3%) | | | 0 (0%) | | 3 (1.3%) | | | 0 (0%) | |
| Non-profit | 244 (4.9%) | 229 (5.1%) | 15 (3.0%) | 16 (5.9%) | 1 (5.6%) | 37 (4.7%) | 4 (4.1%) | 43 (5.1%) | | 0 (0%) | | 23 (5.3%) | | | 2 (4.0%) | | 16 (9.6%) | | | 0 (0%) | | 31 (4.2%) | | | 7 (5.6%) | | 20 (4.6%) | | 0 (0%) | | | 5 (4.0%) | | 0 (0%) | | 22 (4.9%) | | | 1 (1.7%) | | 16 (7.2%) | | | 0 (0%) | |
| Mixed | 817 (16%) | 767 (17%) | 50 (10%) | 75 (28%) | 5 (28%) | 167 (21%) | 13 (13%) | 242 (29%) | | 12 (23%) | | 45 (10%) | | | 1 (2.0%) | | 16 (9.6%) | | | 2 (7.1%) | | 89 (12%) | | | 11 (8.9%) | | 71 (16%) | | 2 (3.5%) | | | 14 (11%) | | 1 (50%) | | 31 (6.8%) | | | 3 (5.1%) | | 17 (7.6%) | | | 0 (0%) | |
| No funding | 1,579 (32%) | 1,436 (32%) | 143 (29%) | 31 (11%) | 0 (0%) | 210 (27%) | 17 (18%) | 50 (6.0%) | | 3 (5.8%) | | 202 (46%) | | | 14 (28%) | | 70 (42%) | | | 7 (25%) | | 339 (46%) | | | 51 (41%) | | 156 (36%) | | 15 (26%) | | | 19 (15%) | | 0 (0%) | | 273 (60%) | | | 31 (53%) | | 86 (39%) | | | 5 (42%) | |
| No data | 563 (11%) | 534 (12%) | 29 (5.8%) | 40 (15%) | 2 (11%) | 26 (3.3%) | 0 (0%) | 252 (30%) | | 14 (27%) | | 7 (1.6%) | | | 0 (0%) | | 10 (6.0%) | | | 0 (0%) | | 66 (8.9%) | | | 8 (6.5%) | | 34 (7.8%) | | 0 (0%) | | | 60 (48%) | | 1 (50%) | | 2 (0.4%) | | | 0 (0%) | | 37 (17%) | | | 4 (33%) | |
| ^1^n (%); Median (Q1, Q3) | | | | | | | | | | | | | | | | | | | | | | | | | | | | | | | | | | | | | | | | | | | | | |
